# Lipid-Binding Aegerolysin from Biocontrol Fungus *Beauveria bassiana*

**DOI:** 10.3390/toxins13110820

**Published:** 2021-11-20

**Authors:** Nada Kraševec, Anastasija Panevska, Špela Lemež, Jaka Razinger, Kristina Sepčić, Gregor Anderluh, Marjetka Podobnik

**Affiliations:** 1 Department of Molecular Biology and Nanobiotechnology, National Institute of Chemistry, SI-1000 Ljubljana, Slovenia; spela.lemez@gmail.com (Š.L.); gregor.anderluh@ki.si (G.A.); marjetka.podobnik@ki.si (M.P.); 2 Department of Biology, Biotechnical Faculty, University of Ljubljana, SI-1000 Ljubljana, Slovenia; anastasija.panevska@bf.uni-lj.si (A.P.); kristina.sepcic@bf.uni-lj.si (K.S.); 3 Biotechnology, Biotechnical Faculty, University of Ljubljana, SI-1000 Ljubljana, Slovenia; 4 Plant Protection Department, Agricultural Institute of Slovenia, Hacquetova 17, SI-1000 Ljubljana, Slovenia; jaka.razinger@kis.si

**Keywords:** beauveriolysin, membrane-attack-complex/perforin (MACPF) protein, pore-forming proteins, entomopathogenic and antagonistic fungi, bioinformatics, ceramide phosphoethanolamine (CPE), lipid binding

## Abstract

Fungi are the most common pathogens of insects and thus important regulators of their populations. Lipid-binding aegerolysin proteins, which are commonly found in the fungal kingdom, may be involved in several biologically relevant processes including attack and defense against other organisms. Aegerolysins act alone or together with membrane-attack-complex/perforin (MACPF)-like proteins to form transmembrane pores that lead to cell lysis. We performed an in-depth bioinformatics analysis of aegerolysins in entomopathogenic fungi and selected a candidate aegerolysin, beauveriolysin A (BlyA) from *Beauveria bassiana*. BlyA was expressed as a recombinant protein in *Escherichia coli*, and purified to further determine its functional and structural properties, including lipid-binding ability. Aegerolysins were found to be encoded in genomes of entomopathogenic fungi, such as *Beauveria*, *Cordyceps*, *Metarhizium* and *Ophiocordyceps*. Detailed bioinformatics analysis revealed that they are linked to MACPF-like genes in most genomes. We also show that BlyA interacts with an insect-specific membrane lipid. These results were placed in the context of other fungal and bacterial aegerolysins and their partner proteins. We believe that aegerolysins play a role in promoting the entomopathogenic and antagonistic activity of *B. bassiana*, which is an active ingredient of bioinsecticides.

## 1. Introduction

Fungi act as decomposers, symbionts or pathogens. They are the most common pathogens of various arthropods and important regulators of their populations. An entomopathogenic fungus acts as a parasite on insects, killing or severely disabling them. After attaching to the insect’s cuticle, conidiospores germinate and form penetrating hyphae that invade the insect’s body, overcome the insect’s innate immune response, and proliferate within it. The victim of the entomopathogenic fungus dies due to vegetative overgrowth and possible release of toxins into the host hemocoel [1]. The fungus feeds on its host, resulting in host death due to dehydration and/or nutrient deficiency [2].

Entomopathogenic fungi may play other roles in nature, including endophytism, antagonism to plant diseases, promotion of plant growth, and root colonization [3,4]. These ecological functions, which are not yet fully understood, suggest the possibility that we may have overlooked their important properties in the development of fungal entomopathogens as biopesticides against insects and other arthropod pests. 

The fungus *Beauveria bassiana* ((Bals.-Criv.) Vuill., 1912) (Hypocreales: Cordycipitaceae) is considered both an entomopathogen and an endophyte. In fulfilling its dual biological role, *B. bassiana* secretes a variety of extracellular enzymes, including chitinases, proteases, lipases, lipoxygenases, amylases, laccases and many others [5,6]. These enzymes are important factors of fungal pathogenicity to arthropods, for their endophytic colonization, and for their popular use as biocontrol agents. Bassiacridin with β-glucosidase, β-galactosidase and N-acetylglucosaminidase activities has already been shown to act as insecticidal proteins [7]. In addition to the enzymes, the fungus also produces a number of metabolites, some of which have already been shown to be insecticidal, such as the simpler-structured dipicolinic acid and oosporein, and the more complex ones, such as beauvericin and its derivatives G1-3, H1-3, isoleucylisoleucylanhydride, cyclo-(L-isoleucyl-L-valine), cyclo-(L-alanyl-L-proline), and bassianolide [6].

The fungus *B. bassiana* is already known as a biopesticide for biological control of some insect pests and as an asymptomatic companion of plants. Several biotic preparations containing the active ingredient of this fungus, which acts as a bioinsecticide, have already been approved. These products are applied as foliar treatments or as soil treatments in which the fungus associates asymptomatically with plants [8]. For example, one of these biotic insecticide preparations is Naturalis (CBC Europe S.r.l.), an oil dispersion containing the active ingredient of *B. bassiana*, strain ATCC 74040, based on live conidiospores. This fungal strain is promising for the control of various disease vectors such as: cabbage whitefly (*Aleyrodes proletella* (Linnaeus, 1758)), aphids (*Aphididae* (Latreille, 1802)), tobacco (cassava) whitefly (*Bemisia tabaci* (Gennadius, 1889)), olive fruit fly (*Bactrocera oleae* (Rossi, 1790)), pear psylla (*Cacopsylla pyri* (Linnaeus, 1758)), mediterranean fruit fly (*Ceratitis capitate* (Wiedemann, 1824)), nut weevil (*Curculio nucum* (Linnaeus, 1758)), click beetle (Elateridae (Leach, 1815)), yellow mite (*Eotetranychus carpini* (Oudemans, 1905)), western flower thrips (*Frankliniella occidentalis* (Pergande, 1895)), lettuce aphid (*Nasonovia ribisnigri* (Mosley, 1841)); fruit tree red spider mite (*Panonychus ulmi* (Koch, 1836)); cherry fruit fly (*Rhagoletis cerasi* (Linnaeus, 1758)), peach tree thrips (*Taeniothrips meridionalis* (Priesner, 1926)); two-spotted spider mite (*Tetranychus urticae* (Koch, 1836)), Thripidae (Stephens, 1829), onion thrips (*Thrips tabaci* (Lindeman, 1889)), *Thrips major* (Uzel, 1895) and greenhouse whitefly (*Trialeurodes vaporariorum* (Westwood, 1856)) [9]. The peer review of this biopesticide risk assessment conducted by European Food Safety Authority (EFSA) found a low risk to birds, mammals, fish, algae, earthworms, soil microorganisms, and non-target plants [10]. However, several data gaps were identified that need to be addressed: (i) the risk of infectivity and pathogenicity to aquatic invertebrates and pollinators when used in the field and greenhouse use; (ii) the risk of infectivity and pathogenicity to honey bee brood and non-target arthropods when used in the field; (iii) the risk to organisms in wastewater treatment when used in the greenhouse; and (iv) there is insufficient information to demonstrate that the toxins/secondary metabolites produced by this fungus do not occur in agroecosystems at significantly higher concentrations than under natural conditions [10]. It is essential to understand the biopesticide activity of this organism as much as possible to avoid or at least understand impacts on non-target organisms.

Rapid advances in nucleotide sequencing technology have accelerated our understanding of entomopathogenic fungi by determining the genomic sequences of several species in the genus *Metarhizium* [11,12], as well as *B. bassiana* [13], *Cordyceps militaris* [14], and *Ophiocordyceps sinensis* [15].

We are interested in proteins of the aegerolysin family, which are characterized by tight beta-sandwich folding, low isoelectric points, low molecular weights, and are stable over a wide pH range [16]. We have identified them in various kingdoms of life, but mainly in fungi and bacteria. Aegerolysins are encoded in the genomes of Dikarya fungi but are absent in some clades, for example Saccharomycotina [17,18].

Some members of the aegerolysin family are known to interact with biological and artificial lipid membranes, either alone or in concert with a membrane-attack-complex/perforin (MACPF)-like partner protein, to form transmembrane pores in artificial and biological lipid membranes [19,20,21,22]. One such an example is a pair of pleurotolysins forming a membrane-embedded pleurotolysin (Ply) pore with a 13-fold symmetry (PDB ID: 4V2T). This pore consists of 26 copies of PlyA, an aegerolysin family protein, and 13 copies of PlyB, a MACPF domain protein [23]. Although the role of aegerolysins and their partner proteins in the producing organism has not been fully elucidated, they may be involved in various processes adapted to the lifestyle of the producing organisms, such as attack and defence against other organisms, ontogenetic development, or cell cycle regulation. The highly identical (94%) proteins PlyA and ostreolysin A6 (OlyA6) from different oyster mushroom (*Pleurotus ostreatus* ((Jacq. ex Fr.) P. Kumm. (1871)) strains have been the best studied aegerolysins to date. They can interact with membrane domains enriched in sphingomyelin in combination with cholesterol, but their high-affinity lipid receptor is ceramide phosphoethanolamine (CPE), an analog of sphingomyelin and the major membrane sphingolipid of invertebrates [16,17,22,24,25,26]. The pore-forming protein complexes composed of *Pleurotus* aegerolysins and their MACPF partner PlyB are able to kill plant pests such as western corn rootworm (*Diabrotica virgifera virgifera* (LeConte, 1868)) and Colorado potato beetle (*Leptinotarsa decemlineata* (Say, 1824)) by targeting the CPE in the invertebrate membranes [27,28].

Thus, entomopathogenic fungi express multiple insecticidal proteins and metabolites, which enable their lifestyle. Our question was whether aegerolysins from *B. bassiana* also interact with CPE and thus contribute to the arsenal of weapons of entomopathogenic fungi. The aim of the present work was to prepare the aegerolysin from the entomopathogenic fungus *B. bassiana* in the recombinant form from *Escherichia coli*, to determine its lipid-binding ability, and to compare it with related proteins from the aegerolysin family with already known lipid affinity. Furthermore, bioinformatics tools were used to describe potential partner proteins.

## 2. Results

### 2.1. Genomes of Entomopathogenic Fungi Encode Aegerolysins

The fungus *B. bassiana* is taxonomically assigned to the subkingdom Dikarya, the phylum Ascomycota, the subphylum Pezizomycotina, the class Sordariomycetes, the order Hypocreales, and the family Cordycipitaceae. The genome of *B. bassiana* consists of 7 or 8 chromosomes and is approximately 34 to 44 Mb in size [29].

Currently, a total of 2040 sequenced genomes of fungal species are deposited in the largest public fungal genome database MycoCosm [30]. According to the taxon classification adopted by MycoCosm, 389 species were classified into the class Sordariomycetes. Among them, we found 105 species that contained protein sequences with the recognized aegerolysin domain PF06355, representing 27% of all species in this clade. In the Sordariomycetes, a total of 115 aegerolysin sequences were identified, indicating that most genomes encode single sequence, but some encode two or even more. For example, the genome of *B. bassiana* [13] as well as the genomes of some de facto entomopathogenic fungi, *M. acridum* ((Driver and Milner) J.F. Bisch., Rehner and Humber, 2009), *M. anisopliae* ((Metschn.) Sorokin, 1883)), *M. robertsii* ((Metchnikoff) Sorokin, 1883)) [11,12], *C. militaris* ((L.) Fr., 1818) [14] and the comparable genome of *P. ostreatus* encode for a single aegerolysin, while *O. sinensis* ((Berk.) G.H. Sung, J.M. Sung, Hywel-Jones and Spatafora, 2007) [15] and the comparable *Aspergillus niger* (van Tieghem, 1867) encode for two [31,32]. Although we identified only one gene locus for the aegerolysin in the whole genome sequence *P. ostreatus* PC9 v1.0 [33], the coding sequences of aegerolysin proteins may vary between strains (Figure S1). For example, the OlyA6 protein is 99% identical to the genomic sequence PlyA(PriA) [34] and PlyA is 95% identical. Similar variation in aegerolysin sequences has been observed previously in some *Altenaria* species and strains [35].

**Figure 1:**
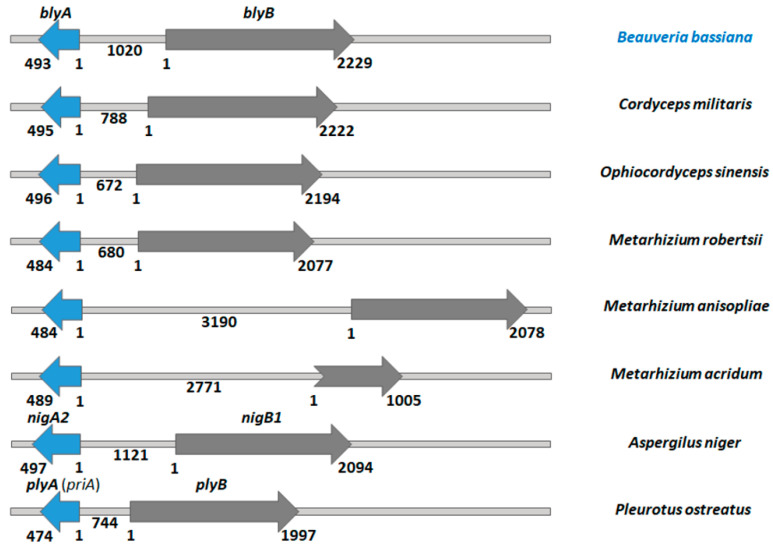
Aegerolysin gene locus in the genomes of the listed fungi. Blue arrows, putative aegerolysin genes; grey arrows, putative membrane-attack-complex/perforin (MACPF)-like genes; *blyA*, beauveriolysin A gene, *blyB*, beauveriolysin A gene B, *nigA2*, nigerolysin A2 gene, *nigB1*, nigerolysin B1 gene, *plyA* (*priA*), aegerolysin gene from *Pleurotus ostreatus* PC9 v1.0 whole genome sequence; *plyB*, pleurotolysin B gene; numbers, size of genes in base pairs. Gene sizes and distances are scaled.

The gene loci of putative aegerolysin and MACPF-like genes in the genomes of some entomopathogenic fungi are schematically shown in Figure 1, in comparison with the genome loci of *A. niger* and *P. ostreatus* studied previously [32]. Identification and annotation of putative MACPF-like proteins is generally more error-prone due to the higher number of introns in these genes. The comparable genomes of *A. niger* and *P. ostreatus* encode gene pairs of aegerolysin and MACPF-like genes, and we also identified such gene pairs in all entomopathogenic species studied. The distances between the two genes vary: From the minimum distance of 672 base pairs (bp) in *O. sinensis*, to 744 bp in the comparative genome of *P. ostreatus*, to 1020 bp in *B. bassiana*, to 1121 bp in the other comparative genome of *A. niger*, and to the maximum distance of 3190 bp in *M. anisopliae*.

### 2.2. Building a Protein Model of Beauveriolysin A

To obtain more information about the putative aegerolysin of *B. bassiana*, BlyA, a protein model was built. The most reliable templates with over 99% confidence were selected from the Phyre^2^ web portal for protein modelling, prediction, and analysis [36]. These four three-dimensional structures of aegerolysins are listed in Table 1. RahU is an aegerolysin from the opportunistic human pathogen *Pseudomonas aeruginosa* ((Schröter 1872) Migula, 1900) (Bacteria, phylum Proteobacteria) [37]. The membrane-binding protein pleurotolysin A (PlyA) (as well as ostreolysin A6 (OlyA6)) is an aegerolysin from the edible mushroom *P. ostreatus* (Fungi, subphylum Agaricomycotina) [23]. The 16 kDa unit of the two-component insecticidal protein (IP-1A) is from *Alcaligenes faecalis* (Castellani and Chalmers, 1919) (Bacteria, phylum Proteobacteria) [38]. The fourth template is the 13.6 kDa insecticidal crystal protein (Cry34Ab1) from *Bacillus thuringiensis* (Berliner, 1915) which belongs to the bacterial phylum Firmicutes [39].

**Figure 2:**
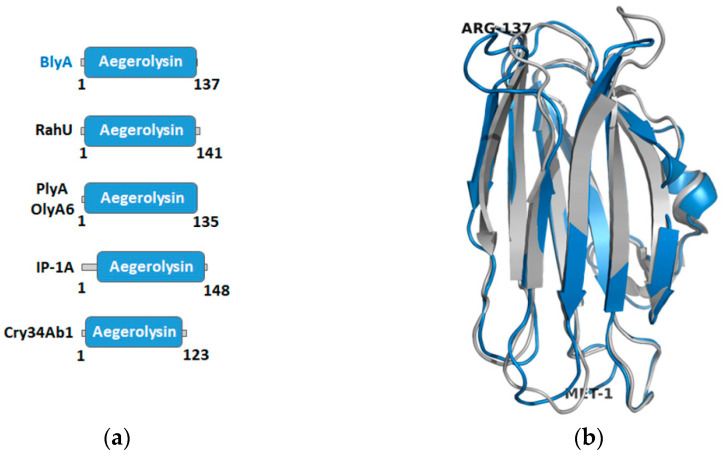
Aegerolysin domains and beauveriolysin A protein model. (**a**) Aegerolysin PF06355 family assigned to the best templates and BlyA (Table 1); BlyA, beauveriolysin A; RahU, aegerolysin from *Pseudomonas aeruginosa*; PlyA, pleurotolysin A; OlyA6, ostreolysin A6; IP-1A, 16 kDa unit of a two-component insecticidal protein; Cry34Ab1, insecticidal crystal protein; blue box, Pfam domain coverage [40]; numbers, number of amino acids in their sequence. (**b**) Protein model of BlyA (in blue) as proposed by Phyre^2^ [36] superimposed with PlyA (PDB ID: 4OEB) [26] (in grey). Confidence in the model: 135 residues (99%) modelled with >90% accuracy. Amino acid at N-and C-terminus of the model are marked. Cartoon representation of the structures using the PyMOL molecular graphics system [41].

The coverage of the BlyA model by the template sequences ranges from 94% to 98%. The amino acid identity of BlyA with the templates ranges from 25% to 36%, which is consistent with the usual identity between different aegerolysins. As expected, the PF06355 aegerolysin family is assigned to BlyA and the templates [40] and almost completely covers the sequences of these proteins (Figure 2a). Finally, to maximize confidence, percent identity and alignment coverage, the three templates RahU (PDB ID: 6ZC1 chain A), PlyA (PDB ID: 4V2T chain 2) and IP-1A (PDB ID: 5V3S chain B) were selected to create the model protein BlyA (Figure 2b). Only one (first) residue was modelled ab initio by Phyre^2^.

Some of the template aegerolysins act as binary toxins (Table 1). Aegerolysin OlyA6, as well as PlyA, are paired with PlyB, which contains a MACPF domain, and forms a heteromeric membrane-embedded pore with 13-fold symmetry [20,23]. Cry34Ab1 is a protein similar in structure to membrane-active proteins, including several cytolysins; its partner is a 43.8 kDa insecticidal crystal protein Cry35Ab1 [42,43]. IP-1A/ IP-1B from *A. faecalis* mimics *B. thuringiensis* by producing a binary protein that acts through a mechanism similar to Cry34Ab1/ Cry35Ab1 [38]. For RahU, there are no data on whether it acts alone or together with a partner protein. From the analysis of the BlyA genomic locus of *B. bassiana* (and other entomopathogenic fungi) (Figure 1) compared to the genomic loci of *A. niger* and *P. ostreatus*, it is expected that aegerolysin BlyA has a protein partner BlyB.

### 2.3. Isolation and Characterisation of Beauveriolysin A

The synthetic *blyA* gene was cloned and expressed in the bacterium *E. coli*. The recombinant BlyA protein was produced as a soluble protein and purified. The observed molecular weight of the purified BlyA (in row 6) (Figure S2a) corresponds well to the theoretically expected Mw size of 14,754 Da. There are two cysteines coded by the protein sequence. To determine whether disulfide bridges were formed in the protein, the mobility of BlyA was analyzed with and without the addition of the reducing agent dithiothreitol (DTT) on the SDS-PAGE gel. No difference in mobility was observed, indicating that no intermolecular disulfide bridges were formed (Figure S2b). In blue native PAGE electrophoresis, which separates proteins by size, shape, and charge, BlyA migrates predominantly uniformly with and without the addition of the reducing agent DTT (Figure S2c). Circular dichroism spectroscopy of the purified sample confirmed the modelling, and showed that BlyA consisted mainly of β-secondary structural elements. Conformational changes due to temperature denaturation were not reversible (Figure S3).

### 2.4. Beauveriolysin A Binds to Ceramide-Phosphoethanolamine or to Sphingomyelin in Combination with Cholesterol

**Figure 3:**
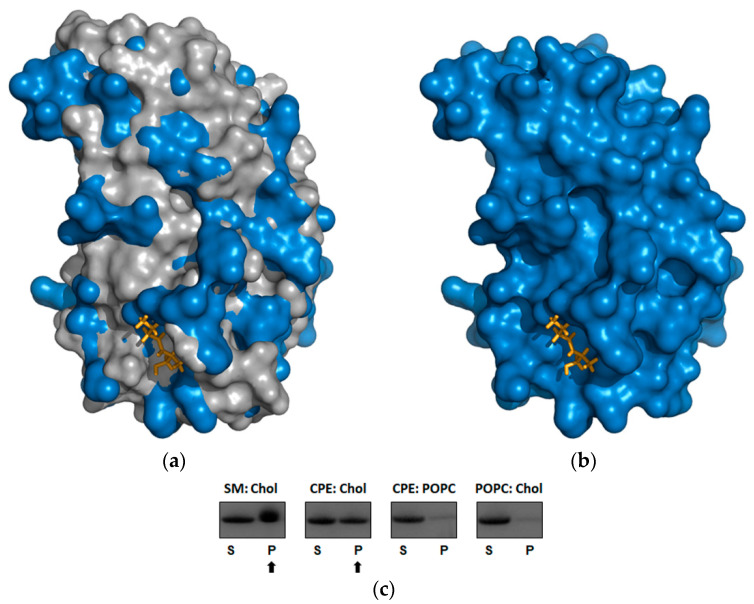
Binding ability of the beauveriolysin A protein to lipids compared to the ostreolysin A6. (**a**) Molecular surface representation of BlyA protein model from this study [36] (in blue) superimposed with the OlyA6 structure (in grey) in complex with the sphingomyelin molecule (partial sphingomyelin model, yellow sticks) (PDB ID: 6MYJ) [26]. (**b**) Same representation of BlyA protein model together with the sphingomyelin only from OlyA6/sphingomyelin complex (PDB ID: 6MYJ) (yellow sticks) is shown. Surface representation of the structures using the PyMOL molecular graphics system [41]. (**c**) Binding of BlyA to multilamellar vesicles containing different lipid mixtures in a 1:1 molar ratio, performed by sedimentation assay (Figure S4). BlyA, beauveriolysin A; OlyA6, ostreolysin A6; SM, sphingomyelin; Chol, cholesterol; CPE, ceramide-phosphoethanolamine; POPC, palmitoyl-oleoyl-phosphatidylcholine; S, supernatant; P, pellet. BlyA in pellet is marked by an arrow.

To infer possible lipid binding, the BlyA protein model was superimposed by the OlyA6 structure plus sphingomyelin (PDB ID: 6MYJ) [26] (Figure 3a,b). To our knowledge, no structure of the aegerolysin complex has been solved with CPE. The binding of BlyA to vesicles containing sphingomyelin/cholesterol (1:1, mol/mol) or CPE/cholesterol (1:1, mol/mol) was confirmed by the sedimentation assay, in which the protein co-sedimented only with vesicles of this composition but not with vesicles consisting of equimolar ratios of palmitoyl-oleoyl-phosphatidylcholine (POPC)/cholesterol or CPE/POPC (Figure 3c and Figure S4).

**Figure 4:**
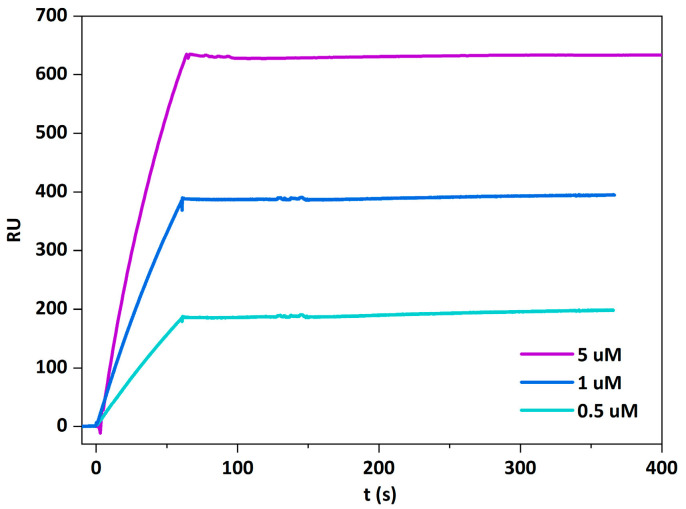
Beauveriolysin A binds to large unilamellar vesicles supplemented with ceramide phosphoethanolamine. Sensorgrams show the kinetics of interaction of BlyA (0.5, 1 and 5 µM) interaction with LUVs (CPE: POPC: Chol, 1:1:1 molar ratio) as determined by the surface plasmon resonance. BlyA, beauveriolysin A; LUVs, large unilamellar vesicles; CPE, ceramide phosphoethanolamine; POPC, palmitoyl-oleoyl-phosphatidylcholine; Chol, cholesterol; RU, response units.

The interaction of BlyA with the invertebrate-specific membrane sphingolipid CPE in complex with cholesterol was confirmed by surface plasmon resonance (SPR), assessing BlyA binding using large unilamellar vesicles (LUVs) composed of equimolar ratios of CPE, POPC, and cholesterol. Binding of BlyA to these vesicles was concentration-dependent and irreversible, as indicated by the SPR sensorgrams during the dissociation phase (Figure 4).

Further analysis of membrane permeabilization of BlyA on bovine erythrocytes showed that BlyA had no hemolytic activity up to 34 µM final concentration (Figure S5). According to the cell viability assays, BlyA alone was also not toxic to Sf9 insect cells up to 5 µM final concentration, which may indicate that this protein requires a protein partner to perform lysis of cells containing sphingomyelin or CPE (Figure S6).

### 2.5. Prediction of Secretion, Localization and Effector Function of Beauveriolysin A

To infer a possible function in fungi, it is helpful to predict the properties of an unknown protein such as its secretion, localization and effector function. Several different prediction tools were used to predict the properties of BlyA compared to the fungal aegerolysins OlyA6 (PlyA) and nigerolysin A2 (NigA2) [44]. Pairwise alignment showed 34% protein sequences identity of between BlyA and OlyA6 (PlyA) and 37% identity between BlyA and NigA2.

The predicted features were not clearly summarized, but most of the inferred features were the same or similar for all three proteins studied. No transmembrane helices (TM) were found using TMHMM [45] and PredαTM [46]. TOPCONS analysis revealed that neither transmembrane regions nor signal peptides were predicted [47]. The PrediSi tool did not predict any signal peptide [48]. Of the ten possible localizations of the deepLoc-1.0 server [49], these three proteins were predicted to be soluble and present in the cytoplasm. The SignalP server predicted secretion pathways other than Sec translocon and cleavage by signal peptidase I (Sec/SPI) [50]. SecretomeP predicted non-classical protein secretion [51]. Phobius [52] predicted a not-cytoplasmatic location, as opposed to the other options of cytoplasm, membrane, or signal peptide. The PredGPI algorithm [53] did not predict a glycosylphosphatidylinositol (GPI) anchor, implying no association with the extracellular leaflet of the plasma membrane. The TargetP tool [54] predicted N-terminal sequences of other types then signal peptides, mitochondrial transit peptides, chloroplast transit peptides, or thylakoid luminal transit peptides. The Wolfpsort prediction suggested that BlyA was probably extracellular [55], which is consistent with the prediction for OlyA6 (PlyA) and NigA2 [44].

There were some differences between the three proteins identified by the PredαTM prediction [46]; there was a possibility that some β-sheet TM regions (20 to 29, 52 to 64, and 83 to 94 amino acids) were present in BlyA, as suggested for NigA2, but not for OlyA6 (PlyA) [44]. In the previous version (EffectorP 2.0), all three proteins were predicted to be effectors. However, according to the new EffectorP 3.0 algorithm, which distinguishes between secreted proteins and secreted effectors in plant pathogenic fungi [56], there was a difference between them: BlyA was proposed as an apoplastic effector, OlyA6 (PlyA) as a cytoplasmic effector, and NigA2 as a non-effector.

### 2.6. Construction of a Protein Model of Beauveriolysin B

The building a model for the possible BlyA partner beauveriolysin B protein (BlyB) was not as successful as for the BlyA protein. Out of 497 amino acid residues, only 190 residues of the BlyB protein, or 38% of the sequence, were modelled with 95.1% confidence by the single template with the highest score. Additional reliable templates were found that covered slightly different regions of the sequence. In total, 292 residues were modelled with multiple templates, corresponding to 59% of the BlyB sequence, with a reliability above 90%, which we considered too low. Ab initio, 203 residues were modelled, making the BlyB protein model very unreliable. The most reliable templates (above 90% confidence) for building the BlyB protein model are listed in Table 2. The coverage of BlyB with the template sequences ranges from 38% to 47%. The amino acid identity of BlyB with the templates ranges from 11% to 16%, which is a rather low identity but common for MACPF-like domain-containing proteins [57].

The Gram-negative insecticidal protein GNIP1Aa was isolated from Proteobacteria *Chromobacterium piscinae* (Kämpfer, 2009) [58]. The macrophage-expressed gene 1 protein (perforin-2) is involved in the human immune system [59,63]. *Photorhabdus luminescens* ((Thomas and Poinar, 1979) Boemare, 1993 emend.) Plu-MACPF is a MACPF/perforin-like protein from the Proteobacteria *P. luminescens* [61]. The murine complement component C9 [60] and the human complement component C8 alpha chain are involved in the immune system [62].

No data are available for the partner proteins of GNIP1Aa and Plu-MACPF. MPEG-1 is thought to function alone, in contrast to proteins C8 and C9, which are involved in the assembly of a heterooligomeric protein-membrane attack complex (MAC) [59,60,62,63].

**Figure 5:**
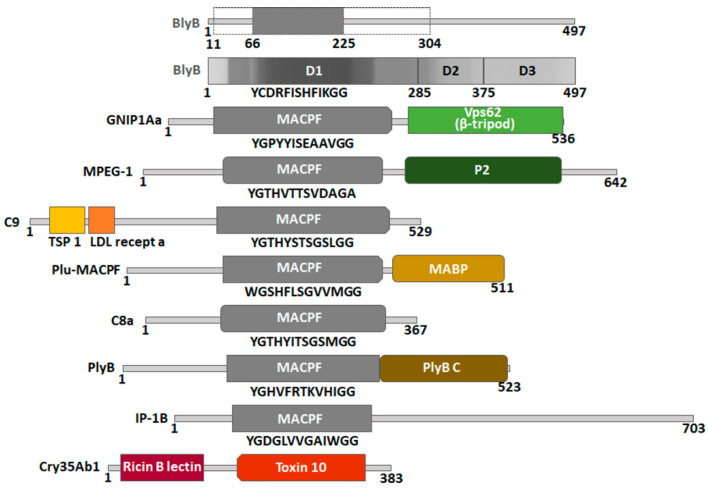
Assigned domains to the best templates for the beauveriolysin B protein model and to the binary toxin partners of beauveriolysin A. The Phyre^2^ model templates are listed in Table 2, the binary toxin partners in Table 1, and the position of the domains in Table S1. BlyB, beauveriolysin B; GNIP1Aa, Gram-negative insecticidal protein; MPEG-1, macrophage-expressed gene 1 protein (perforin-2); C9, complement component C9; Plu-MACPF, *Photorhabdus luminescens* MACPF protein; C8a, complement component C8 alpha chain; PlyB, pleurotolysin B; IP-1B, insecticidal two-component protein, 77 kDa unit; Cry35Ab1; 43.8 kDa insecticidal crystal protein. Domains were assigned from EMBL-EBI Pfam [40,64] and some additional domains from InterPro [65] or from reference [63]. MACPF, membrane attack complex/perforin (PF01823); Vps62, vacuolar protein sorting-associated protein 62 (PF06101); β-tripod, β-tripod domain [58]; P2, peripheral domain P2 TSP 1, thrombospondin type 1 domain (PF00090); Ldl recept a, low-density lipoprotein receptor class A domain (PF00057); MABP, MVB12-associated β-prism domain (IPR023341); PlyB_C, pleurotolysin B C-terminal domain (PF18684)**;** ricin_B_lectin, ricin-type β-trefoil lectin domain (PF00652); toxin 10, insecticidal crystal toxin P42 (PF05431). Numbers, size of proteins in kDa. Typical 13-amino acid signature for the MACPF domain. Dashed lines, part of the BlyB protein model created according to the templates, the outer regions *ab inicio*. D1, D2, D3, domains as found by FUpred analysis [66] and hit coverage by PHmmer [67].

Pfam domains were assigned to BlyB and the best model templates of BlyB: GNIP1Aa, MPEG-1, C9, Plu-MACPF and C8a and the binary partners of aegerolysin templates: PlyB, IP-1B and Cry35Ab1 (Figure 5) [40,64]. Some protein domains were identified using the InterPro tool [65] or the literature [63]. All proteins in this group were assigned to the MACPF domain PF01823, which was confirmed by the 13-amino acid signature Y/F-G-X2-F/Y-X6-G-G typical of this domain [57]. GNIP1Aa has two domains; in addition to the MACPF domain, there is also a partial Vps62 domain (named after vacuolar protein sorting-associated protein 62) PF06101 at the C-terminus. MPEG-1 a has a peripheral P2 domain characterized at the C-terminus [59,63]. Two additional domains were identified in the C9 protein template sequence: TSP1_spondin (spondin-like TSP1 domain) PF00090 and Ldl recept a (low-density lipoprotein receptor class A domain) PF00057 at the N-terminus. Plu-MACPF has another additional MABP domain (the name stands for MVB12-associated β-prism domain) IPR023341at the C-terminus. In addition to the MACPF domain, PlyB, a binary partner of OlyA6 or PlyA, has an additional C-terminal domain-PlyB_C PF18684 [23]. For IP-1B, the binary partner of IP-1A, the MACPF domain was found to be insignificant only.

The only exception among the listed proteins is the binary protein partner Cry35Ab1, which is not assigned a MACPF domain. It was replaced by the C-terminal domain toxin 10 (PF05431) (family of insecticidal crystal toxins of *Bacillus*, named after the insecticidal crystal toxin P42) [40,64]. Cry35Ab1 also has an additional N-terminal domain, the ricin-type β-trefoil lectin domain (PF00652) (classified as insignificant only) [65].

No Pfam domain at all was assigned for BlyB. However, part of the MACPF domain was included from the modeling templates, at least-between 66 and 225 amino acids (123 total) and at most-between 11 and 304 amino acids (293 total). The prediction tool FUpred [66] suggested three different domains in this protein: D1 domain (between 1 and 285 amino acids) corresponding to the MACPF-like N-terminus, and additionally two domains at the C-terminus, D2 (between 286 and 374 amino acids) and D3 (between 375 and 497 amino acids). 

## 3. Discussion

### 3.1. The Genomes of Entomopathogenic Fungi Encode Binary Toxins

The genomes of *A. niger* and *P. ostreatus* encode gene pairs for aegerolysin and MACPF-like genes, and such pairs were also identified in all entomopathogenic species studied. From the positions of the genes in the genome of *A. niger*, it is evident that the *nigA2* and *nigB1* genes form a bidirectional gene pair with 5′–5′ orientation, as do the *plyA* and *plyB* genes in the genome of *P. ostreatus* [32]. A similar situation was found in the genomes of experimentally detected entomopathogenic fungi of *B. bassiana* [8] and in the genomes of *C. militaris*, *M. acridum*, *M. anisopliae*, *M. robertsii*, and *O. sinensis*.

### 3.2. Beauveriolysin A

Of the four templates for the protein model of BlyA (Table 1), only one aegerolysin belongs to a fungal species from the subphylum Basidiomycota, a sister subphylum of Ascomycota, which includes the fungus *B. bassiana*. The other three templates are bacterial but taxonomically belong to two different phyla Proteobacteria and Firmicutes.

In the sedimentation assay with MLVs, we found that BlyA binds to an invertebrate-specific sphingolipid CPE or sphingomyelin in combination with cholesterol. This is consistent with previously discovered properties of several aegerolysins of fungal (*Pleurotus* sp. and *A. niger*) and bacterial (*P. aeruginosa*) origin. When MLVs were composed of CPE and cholesterol in equimolar ratio, co-sedimentation was observed for OlyA6 from *P. ostreatus*, PlyA2 and erylysin A (EryA) from *P. eryngii* [28], NigA2 from *A. niger* [32] and RahU from *P. aeruginosa* [37]. In the case of MLVs composed of sphingomyelin and cholesterol (1:1, mol:mol), co-sedimentation was observed only for some of the above-mentioned aegerolysins: in addition to for BlyA also for OlyA6 and PlyA2 [28].

*Pleurotus* aegerolysins, NigA2 and RahU have been shown to bind to CPE in insect Sf9 cells (pupal epithelial ovarian cells of the moth *Spodoptera frugiperda* (J. E. Smith, 1797) or in insect tissues, as well as to the bloodstream form of *Trypanosoma brucei* (Plimmer and Bradford, 1899) [22,28,32,37,68]. In addition, the OlyA6/PlyB, PlyA/PlyB and EryA/PlyB complexes can effectively permeabilize insect cells of selected Coleoptera pests, the western corn rootworm and the Colorado potato beetle [28]. The columnar epithelium of the midgut wall of western corn rootworm larvae fed OlyA6/PlyB showed vacuolization of the cell cytoplasm, swelling of the apical cell surface into the intestinal lumen, and delamination of the basal lamina underlying the epithelium. Moreover, membrane interactions of the OlyA6/PlyB complex formed multimeric transmembrane pores on lipid vesicles composed of artificial lipids containing CPE and brush border membrane vesicles of western corn rootworm, suggesting that the molecular mechanism of the insecticidal action of OlyA6/PlyB is based on specific interactions of OlyA6 with CPE and the resulting formation of transmembrane pores in the insect midgut [69]. Further toxicity feeding tests with *Pleurotus* aegerolysin/PlyB complexes were performed with two ecologically important non-target arthropod species: the woodlouse (*Porcellio scaber* (Latreille, 1804)) and the honeybee (*Apis mellifera* (Linnaeus, 1758)). None of the *Pleurotus* aegerolysin/PlyB complexes were toxic to isopods, but OlyA6/PlyB and PlyA2/PlyB were toxic to honeybees, whereas no toxicity to ecologically beneficial arthropods was observed for EryA/PlyB at the concentrations tested [70].

Expression and isolation of the identified BlyB will lead to further clarification of the function of the possible binary BlyA/PlyB toxin from *B. bassiana*.

### 3.3. Templates for the Beauveriolysin A and B Protein Models and Their Protein Partners Function as Insecticidal or Immunological Protein Complexes

Comparing BlyA and BlyB with the proteins of similar structure, we identified several proteins: PlyA, IP-1A, Cry34Ab1, and RahU (aegerolysins), and PlyB, IP-1B, Cry35Ab1, GNIP1Aa, Plu-MACPF, MPEG-1, C8a, and C9 (aegerolyin partner or template proteins). The proteins in each group not only share a similar structure, but some also share a common function as insecticidal or immune systems proteins.

The pore-forming protein complexes of OlyA6/PlyB, PlyA2/PlyB, and EryA/Ply kill plant pests such as western corn rootworm and Colorado potato beetle [28]. Other examples of insecticidal complexes that are selective against the western corn rootworm are IP-1A/IP-1B of *A. faecalis* and Cry34Ab1/Cry35Ab1 of *B. thuringiensis* [38,39]. It should be noted that IP-1B is significantly larger (703 amino acids) than Cry35Ab1 (385 amino acids). Although the *A. faecalis* species emulates *B. thuringiensis* in the production of a binary protein, no similarity was found between the sequences of Cry35Ab1 and IP-1B. In contrast, IP-1B was found to contain a MACPF-like domain like PlyB instead of the toxin 10 domain as in Cry35Ab1. GNIP1Aa is a Gram-negative insecticidal protein that is specifically toxic to western corn rootworm larvae when they feed on it [58]. The origin of Plu-MACPF is *P. luminescens*, a lethal pathogen of the insects [61]. The toxin RahU is the product of the bacterium *P. aeruginosa* [37], a known ubiquitous opportunistic bacterial human pathogen that infects a remarkably wide range of species, vertebrates, plants, and also insects; it is an important drug-resistant pathogen and has shown innate potential to promote plant growth and biological control in vitro [71,72].

Other listed proteins act as immune components, which is not surprising since it is known that human complement membrane attack proteins share a common fold with bacterial cytolysins. MPEG-1 is involved in the human immune system [59,63] and acts in the phagolysosome to damage engulfed microbes. MPEG1 is thought to form pores in target membranes, but its exact mode of action is still unknown. The C8 and C9 proteins are involved in the formation of a heterooligomeric protein-membrane attack complex (MAC). Assembly begins with local production of C5b and binding of C6 to form a soluble, noncovalently bound complex. Subsequent binding of C7 results in a complex that binds to the target cell but does not fully penetrate the membrane. After binding to the membrane, this complex recruits C8 into the complex. In the final step, the complex binds a C9, which initiates the recruitment and self-polymerization of additional C9 proteins to form a transmembrane pore [59,60,62,63]. The listed aegerolysins (Table 1) share the same three-dimensional fold to BlyA from entomopathogenic fungus and, together with partner proteins, mostly have an insecticidal or immunological function, which may indicate the future direction of BlyA function research.

### 3.4. Function of Additional Domains in Templates or Partner Proteins

Proteins generally consist of one or more functional regions commonly referred to as domains. Different combinations of domains lead to the diversity of proteins found in nature. Therefore, identification of domains in proteins can provide insights into their function [73]. In addition to the MACPF domain, which serves as a pore-forming protein component, the listed proteins (Figure 5) have another domain that appears to function as an accessory recognition domain for preferably lipids, but also proteins or carbohydrates at target membranes, or for cell recognition. In addition to aegerolysins, these accessory domains seem to contribute to the fine selection of the target.

The accessory domains were mostly located at the C-terminus of the proteins (Figure 5). For the partial Vps62 domain of GNIP1Aa, it was proposed to split it into two parts and redefine one part into a β-tripod domain with D-X-G-S/T-G-X3-D recognition sequence. The β-tripod domain plays the role of an additional target recognition domain [58]. The P2 domain is unique to MPEG-1 and is evolutionarily conserved in all taxa that have a MPEG-1 homolog. Recent structural and functional studies suggest that the P2 domain makes contact with target membranes. It has also been found that the P2 domain preferentially binds liposomes containing negatively charged lipids [59,63]. Plu-MACPF has another additional MABP domain at the C-terminus, which has an internal repeat structure of three homologous segments-β-prism domains-with an internal 3-fold symmetry. It has been suggested that the MABP domain has a membrane-associated function. It is plausible that the eukaryotic MABP domains serve as adaptors that support the binding of other associated domains of the same polypeptide to vesicular membranes [61,74]. PlyB also has another C-terminal domain-PlyB_C [23], a trefoil β-sheet-rich domain that sits on top of the PlyA dimer when the two interact to form relatively small and regular pores in liposomes.

Three N-terminal domains were also listed (Figure 5). A number of proteins involved in the complement pathway contain one or more copies of the TSP 1 domain repeat. It is involved in cell-cell interaction, inhibition of angiogenesis, and apoptosis [75,76]. Ldl recept a encodes a class of structurally related cell surface receptors that serve distinct biological functions in different organs, tissues, and cell types. The function most commonly associated with this evolutionarily ancient family is cholesterol homeostasis [77]. The ricin-type B-trefoil lectin domain contains β-trefoil sequences reminiscent of the carbohydrate-binding domain of the ricin B subunit. In some cases, it has been shown to bind simple sugars, such as galactose or lactose [78,79,80].

Of the three inferred domains of BlyB, D1 at the N-terminus matched well with the MACPF-like domain. The hit coverage of D2 and D3 suggested that they are less abundant in protein database and therefore more specific than the D1 domain. As indicated by the accessory domains listed above, two additional domains, D2 and D3, together with the aegerolysin BlyA may be responsible for fine-tuning the selection of the target.

### 3.5. Localization of Aegerolysins in Fungi

Several different prediction tools such as secretion, localization and effector function were applied to BlyA to infer localization/function in fungi. The prediction results were compared with the two aegerolysins OlyA6 (or PlyA) from *P. ostreatus* and NigA2 from *A. niger*. Although the pairwise sequence alignments revealed only 34% (for both) and 37% identity, respectively, most of the predicted properties were similar for all three proteins tested.

Secretion of NigA2 has already been studied using secretion prediction tools and Western blotting, and subcellular localization was further investigated by immunocytochemistry and live-cell imaging. In *A. niger*, the leaderless NigA2 protein is uniformly distributed throughout the cytoplasm of fungal hyphae and reaches the cell exterior by unconventional protein secretion [44].

It is expected that the leaderless BlyA protein from *B. bassiana* has a similar distribution to NigA2 in hyphae and acts as an effector protein after unconventional protein secretion. Characteristics observed for aegerolysins such as: (i) small size, solubility, and resistance to extreme pH and temperature; (ii) the absence of a signal sequence for classical secretion; (iii) the low number of cysteine residues and disulfide bridges; and (iv) the lack of glycosylation, have also been proposed for some groups of fungal defense proteins, including actinoporin-type lectins, beta-trefoil-type lectins, beta-trefoil-type chimerolectins, beta-propeller-type lectins and galectins. These exceptional properties make such fungal proteins attractive for crops protection against plant pests and for veterinary and human medicine against parasites [81].

### 3.6. Future Research Directions to Improve the Properties of Biopesticides

Entomopathogenic fungi have been developed as environmentally friendly alternatives to chemical insecticides for biocontrol of agricultural pests and disease vectors. In addition to strain selection [82], genetic engineering is also among the options for strain improvement of fungal biopesticides for specific control of insect pests [83,84,85,86,87]. Recombinant entomopathogenic agents with better fungal biopesticidal properties can be achieved by introducing (i) pathogen-derived genes to improve virulence, (ii) insect proteins, (iii) genes from insect predators and other insects, or (iv) invented proteins.

For example, *B. bassiana* is widely used to kill mosquito larvae and adults in the laboratory and field, but its virulence is slow. The exogenous cytolytic δ-endotoxin Cyt2Ba from the entomopathogenic bacterium *B. thuringiensis* was introduced into *B. bassiana* to increase its virulence against *Aedes* (Meigen, 1818) mosquitoes [88]. Cyt2Ba is found in Bt crystals composed of one or more proteins, also known as δ-endotoxins (Cry- and Cyt-toxins), which are very specific for the target insect but safe for other non-target organisms [89]. Their members possess insecticidal, hemolytic, and cytolytic activities through pore formation and are attract attention because they can be used as vehicles for targeted membrane destruction [90]. Overexpression of *B. bassiana* own genes, *blyA* and *blyB*, can be considered as the obvious candidates to enhance virulence (as suggested in (i)); further studies are needed to confirm their suitability. Aegerolysins such as BlyA or synthetic analogs may be further developed in order to alter their affinity for their lipid targets. For example, a single point mutation has already been shown to increase the ability of OlyA6 to bind free sphingomyelin [26] (iv). Alternatively, aegerolysins could direct various toxins (other than MACPF-like domain containing proteins) to CPE-containing membranes (iv).

### 3.7. Future Research Directions to Improve Antagonism against Plant Diseases

The fungus *B. bassiana* is not only an entomopathogen, but also an endophyte, an antagonist against plant diseases, a plant biostimulant, and a root colonizer. Looking at the list of organisms containing CPE as the dominant membrane sphingolipid (Table 3), it is possible to identify a kingdom of organisms that includes many important plant pathogens-Oomycota with the species *Pythium ultimum* (Trow, 1901), *Phytophthora infestans* ((Mont.) de Bary, 1876) and *P. capsici* (Leonian, 1922).

Another filamentous fungus used for biological control of plant diseases is *Trichoderma atroviride* (Bissett, 1984), which is widely distributed in the rhizosphere and has been extensively studied for its ability to parasitize other fungi and compete with harmful plant microorganisms. The versatility of *Trichoderma*’s interactions is due to its ability to interact between and across different kingdoms of organisms [91,92]. The process of mycoparasitism and competition appears to be very complex and involves not only plant pathogenic fungi but also Oomycetes such as *P. ultimum* [93], *Phytophthora cinnamomi* (Rands, 1922) [94] and *Phytophthora nicotianae* (Breda de Haan, 1896) [95]. To illustrate these processes, more and more tripartite studies on the relationships between plant, *Trichoderma* and phytopathogen or plant, *Trichoderma* and insect have been carried out, and several molecular interactions have already been revealed. Recently, the functionally characterized aegerolysin gene *agl1* of *T. atroviride*, has also shown a role in antagonism and conidiation [96], although its lipid specificity has not yet been elucidated.

## 4. Conclusions

Beauveriolysin A (BlyA) is the aegerolysin of the known entomopathogenic and also antagonistic fungus *B. bassiana*. Structural modelling suggests that it has a similar three-dimensional fold to other aegerolysins from fungi or bacteria. BlyA showed the ability to bind specifically to lipid vesicles containing cholesterol in combination with sphingomyelin or CPE. The latter is the major sphingolipid in the lipid membranes of various invertebrates, including insects. However, BlyA itself is neither hemolytic to bovine erythrocytes nor toxic to Sf9 insect cells. Given its similarity to other fungal pore-forming two-component proteins, we investigated whether the genome of *B. bassiana* also encodes its pore-forming partner protein, termed “component B”. We found a candidate BlyB, but the possible formation of pores together with BlyA remains to be confirmed. It is possible that similar protein pairs are encoded by several entomopathogenic fungi of the class of Sordariomycetes. In addition, several proteins that served as templates for building a protein model in this study, or possible binary toxin partner proteins, or organisms from which these proteins are derived, also share insecticidal (or immunological) properties. We believe that the beauveriolysins of *B. bassiana* may belong to a set of multiple insecticidal proteins and metabolites that enable entomopathogenic fungi to have their specific lifestyle.

## 5. Materials and Methods

### 5.1. Comparative Genomics, Prediction and Phylogenetic Methods

Mining for aegerolysins from fungi was performed on the web portal *MycoCosm* [30,97]. The database was searched for domain PF06355 named aegerolysin. The genomes of *Beauveria bassiana* ARSEF 2860 [13], *Cordyceps militaris* CM01 [14], *Metarhizium acridum* CQMa 102, *Metarhizium anisopliae* ARSEF 549, *Metarhizium robertsii* ARSEF 23 [11,12], *Ophiocordyceps sinensis* IOZ07 [15], *Pleurotus ostreatus* PC9 v1.0 [33] and *Aspergillus niger* CBS 513.88 [31] were screened for adjacent aegerolysin genes.

Protein models were built using the *Phyre^2^* web portal for protein modelling, prediction, and analysis [36]. Visual representation of the structures was done using *PyMOL*, version 1.7.2.1 [41].

The *Pfam* database is a large collection of protein families, each represented by multiple sequence alignments and hidden Markov models (HMMs). Pfam version 34.0 (March 2021) was created at European Bioinformatics Institute using a sequence database called Pfamseq, which is based on UniProt version 2020_06 [40,64,73]. HMMER Biosequence analysis implements methods that use probabilistic models called profile HMMs; it can be used in conjunction with a profile database, such as Pfam or many of the databases that participate in Interpro. In *PHmmer* (EMBL-EBI HmmerWeb version 2.41.1), a search for protein sequence *versus* protein sequence database UniProtKB was performed to obtain hit coverage [67,98]. *FUpred* is a contact-map based domain prediction method that uses a recursion strategy to detect domain boundaries based on the predicted contact map and secondary structure information; the method has excellent performance in detecting discontinuous domain boundaries [66,99].

Analysis of amino acid conservation between the protein sequences of BlyA, OlyA6, PlyA and NigA2 was performed using *ClustalW* [100].

The *DeepLoc-1.0* server predicts the subcellular localization of eukaryotic proteins using the neural networks algorithm trained on Uniprot proteins with experimental evidence for subcellular localization. The algorithm uses only the sequence information of the protein to perform the prediction [101]. Eukaryotic proteins distinguish between ten different subcellular localizations: nucleus, cytoplasm, extracellular, mitochondrion, cell membrane, endoplasmic reticulum, chloroplast, Golgi apparatus, lysosome/vacuole and peroxisome [49]. *EffectorP* has been trained to distinguish secreted proteins from secreted effectors in plant pathogenic fungi and oomycetes. The new version EffectorP 3.0 extends the predicted effector repertoires beyond small, cysteine-rich secreted proteins in fungi and RxLR motif containing secreted proteins in oomycetes through two machine learning models: one trained on apoplastic effectors and one trained on cytoplasmic effectors [56,102]. *Phobius* predicts signal peptides and transmembrane topology from the amino acid sequence of proteins. Localization is distinguished between cytoplasm, non-cytoplasm, membrane or signal peptide [52,103]. Default settings were used. Several eukaryotic proteins associated with the extracellular leaflet of the plasma membrane carry a GPI anchor. The *PredGPI* prediction method is able to efficiently predict both the presence of the GPI-anchor and the position of the ω-site, by coupling a Hidden Markov Model and a Support Vector Machine [53,104]. *PrediSi* predicts the signal peptide and cleavage site in eukaryotes and bacteria based on a position weight matrix improved by a frequency correction that accounts for amino acid biases in the proteins considered. The software was trained using sequences from the SwissProt database [48,105]. The organism group was set to eukaryotic. The *PredαTM* and *PredβTM* algorithms predict α- or β-transmembrane segments of integral membrane proteins by a support vector machine classifier trained on sequence data of transmembrane proteins with known structures. The algorithms predict likely transmembrane regions based on amino acid adjacency frequency and position-specific preference of amino acids in transmembrane regions [46,106,107]. *SecretomeP* predicts non-classical protein secretion ab initio. The method is based on obtaining information about various post-translational and localizing proteins involved in the final prediction of secretion [51,108]. *SignalP 5.0* is based on a combination of different neural networks. It predicts the presence and location of three types of signal peptides and cleavage sites in eukaryotes: secretory signal peptides transported by the Sec-translocon and cleaved by signal peptidase I (Sec/SPI); lipoprotein signal peptides transported by the Sec-translocon and cleaved by signal peptidase II (Sec/SPII); and Tat signal peptides transported by the Tat-translocon and cleaved by signal peptidase I (Tat/SPI) [50,109]. Standard mammalian parameters were used for the prediction tool. *TargetP* predicts subcellular localization for eukaryotic proteins based on the presence of N-terminal sequences: signal peptides, mitochondrial transit peptides, chloroplastic transit peptides, or thylakoid luminal transit peptides [54,110]. The tool was set to non-plant parameters. *TMHMM* predicts the presence of transmembrane helices in proteins [45]. *TOPCONS* is a tool for consensus prediction of signal peptides and membrane protein topology. The TOPCONS2 web server has been used for combined predictions [47,111]. *Wolfpsort* predicts protein subcellular localization based on sorting signals, amino acid composition and functional motifs based on conversion to numerical functions. After conversion, a simple k-nearest neighbour classifier is used for prediction. Wolfpsort was set to standard parameters for fungi [55,112].

### 5.2. Cloning of Beauveriolysin A

The synthetic construct encoding beauveriolysin *blyA* (GenScript, Piscataway, NJ, USA) was optimised for expression in *E. coli*. It contained the nucleotide sequence of the *blyA* gene (hypothetical protein BBA_02700) from the publicly available genome of *B. bassiana* ARSEF 2860 [13] and the nucleotide sequence encoding the Tev protease cleavage site (3′-end) and three restriction sites for the restriction enzymes *Nde*I (5′-end), *Bam*HI and *Xho*I (3′-end). Using the restriction enzymes *Nde*I and *Xho*I, this construct was inserted into the pET-21c(+) vector to produce the C-terminal His-tagged protein BlyA. The final construct was sequenced. The same cloning strategy was previously used for recombinant NigA2 production [17].

### 5.3. Expression and Isolation of Recombinant Beauveriolysin A

The plasmid vector containing the construct encoding recombinant BlyA was transformed cells by heat shock at 42 °C into BL21(DE3)-competent *E. coli*. After an initial 1-h incubation in Luria–Bertani medium (1% (*w*/*v*) tryptone, 0.5% (*w*/*v*) yeast extract, 1% (*w*/*v*) NaCl, 1.5% (*w*/*v*) agar) at 37 °C with agitation, the transformed cells were plated onto selective Luria–Bertani medium containing 0.1 g/L ampicillin and grown overnight at 37 °C. Liquid culture of the transformed bacterial cells was first prepared in a small volume (e.g., 20 mL of selective Luria–Bertani medium containing 0.1 g/L ampicillin), and the next day, 10 mL of it was used to inoculate a larger volume of the same medium for protein production (e.g., 1 L). When the culture reached an OD600 of 0.5, expression of the *blyA* gene was induced by adding 1 mL of 0.5 mM isopropyl β-D-1-thiogalactopyranoside. The transformed cells were grown in liquid culture, at 37 °C and rotation speed of 180 rpm. After induction, cells were grown for 29 h at 20 °C and 180 rpm. The bacterial biomass was then collected by centrifugation (10 min, 7808× *g*, 4 °C) and stored at −20 °C.

The biomass was re-suspended in lysis buffer (50 mM NaH_2_PO_4_ ×2H_2_O, 300 mM NaCl, pH 7.5) containing protease inhibitors (0.5 mM phenylmethylsulfonyl fluoride, 1 mM benzamidine) and enzymes (0.5 mg/mL lysozyme, 5 U/mL benzonase nuclease, 20 µg/mL RNase). Bacterial cells were disrupted for 10 min by sonication (pulsed, 1 s on, 2 s off; amplitude 38%), and the homogenate obtained was centrifuged (45 min, 50,000 rpm 47,850× *g*, 4 °C). The recombinant BlyA was present as a soluble protein and was therefore isolated from the centrifugation supernatant by nickel affinity chromatography (Ni-NTA Superflow nickel-charged resin; Qiagen, Hilden, DE) according to the manufacturer’s instructions. After the initial elution of the unbound proteins with lysis buffer, the nonspecifically bound proteins were eluted with wash buffer (lysis buffer containing 25 mM imidazole), and then the bound His-tagged recombinant protein BlyA was eluted with elution buffer (lysis buffer containing 300 mM imidazole). The high imidazole concentration was reduced, and the recombinant BlyA was processed by laboratory produced Tev protease during dialysis in lysis buffer overnight inside the 6000 Da to 8000 Da molecular weight cut-off membrane. The next day, an additional nickel affinity chromatography step was performed, and the separated product was collected in the unbound fraction.

This lysis buffer was exchanged by dialysis for the erythrocyte buffer (20 mM Trizma base, 140 mM NaCl, pH 7.4) or the phosphate buffer (50 mM NaH_2_PO_4_ × 2H_2_O pH 7.5, 300 mM NaCl) and the sample was concentrated by centrifugal filtration (NMWL10; Amicon Ultra 15, Merck, Darmstadt, DE). The recombinant BlyA was stored in the erythrocyte buffer or phosphate buffer containing 5% glycerol at −70 °C.

### 5.4. SDS-PAGE-Analysis and Blue Native Electrophoresis

Commercially available Bis-Tris gels with cross-linking rate of 4–12% (NuPAGE Bis-Tris Protein Gels, Thermo Fisher Scientific, Waltham, MA, USA) were used for SDS-PAGE analysis. DTT reducing agent (0.5 M) was added at one-tenth of the final sample volume or replaced with MilliQ.

For blue native electrophoresis 4x NativePAGE Sample Buffer was added to BlyA and the sample was loaded onto a native electrophoresis gel (Invitrogen BlueNativePAGE Bis-Tris Gel 3-12%, Thermo Fisher Scientific, Waltham, MA, USA) without denaturation. Electrophoresis was performed at 4 °C. The gel was decolorized with Milli Q to detect protein staining. After complete decolorization, the gel was stained with silver.

### 5.5. Circular Dichroism Spectroscopy

BlyA, which was stored in phosphate buffer containing 5% glycerol, was used for the procedure. To reduce the salt and protein concentration in the sample, 50 μL of protein with a concentration of 2.37 mg/mL was diluted ten times with 0.01 M NaH_2_PO_4_ buffer, pH 8. The absorbance was measured in the far UV spectrum (200 to 250 nm). The BlyA protein was heated stepwise from 20 to 100 °C and finally cooled to 20 °C. To avoid the contribution of the buffer to the absorbance, we also prepared a blank sample containing only a dilution of phosphate buffer with 5% glycerol in the same ratio as the sample. At each temperature, the instrument repeated the measurement 5 times to avoid errors. The measurement was repeated twice.

### 5.6. Protein Sedimentation Assay with Multilamellar Lipid Vesicles

Binary multilamellar vesicles were prepared as previously described [113]. These were composed of equimolar ratios of different lipids, including: CPE (Matreya, State College, PA, USA), POPC, porcine brain sphingomyelin, and wool grease cholesterol (all from Avanti Polar Lipids, Alabaster, AL, USA). Each lipid was weighed and dissolved in stock solutions containing chloroform (except CPE in stock solution 9:1 = chloroform:methanol + 2 μL Milli Q). Appropriate stock solutions were pipetted into glass flasks to obtain the desired ratio of each lipid. The organic solvents were evaporated on the rotary evaporator. The film formed at the bottom of the flasks was then swollen in 500 μL of the desired buffer (the same in which the protein is kept, without glycerol). We then added glass beads and vortexed to form vesicles, until no lipid film was visible on the flask walls. 20 μL of the different vesicles at a concentration of 5 mg/mL were pipetted into two micro-centrifuges and 20 μL of BlyA in phosphate buffer at a final concentration of 34 μM was added. Sedimentation assay was performed as previously published [28]. These samples were then subjected to SDS-PAGE electrophoresis along with the originally stored sediments, to determine whether BlyA was present in the original sediment with vesicles (membrane-bound BlyA) or in the original supernatant (free BlyA). Pellets were diluted in equal volumes of the non-reducing SDS sample buffer (200 mM Tris-HCl, pH 8, 5% (*w*/*v*) SDS, 2 mM EDTA, 0.1% (*w*/*v*) bromophenol blue), heated to 100 °C for 5 min, and applied to homogeneous 12% acrylamide gels. Proteins in the gels were stained with SimplyBlue SafeStain (Thermo Fisher Scientific, Waltham, MA, USA). The experiment was repeated twice.

### 5.7. Surface Plasmon Resonance

Large unilamellar vesicles (LUVs) with the composition CPE:POPC:Chol (1:1:1) were prepared as published [37]. Binding of the recombinant BlyA protein (stored in phosphate buffer containing 5% glycerol) was assayed at a final concentration of 5 μM, 1 μM, and 0.5 μM. The interactions between BlyA and LUVs were monitored with a SPR-based refractometer (Biacore T100) using the L1 chip [114]. LUVs (100 µg lipids/mL; 600-s injection at 2 µL/min) were immobilized with running buffer (10 mM HEPES, 150 mM NaCl, pH 7.4). LUVs were bound to the second flow cell of the L1 sensor chip, with a response of approximately 8000 response units (RU). The first flow cell was left empty and used for nonspecific binding of proteins to the dextran matrix, which was minimized by a 1-min injection of 0.1 mg/mL bovine serum albumin at a flow rate of 30 μL/min. To test the interactions between BlyA and the LUVs, the BlyA was dissolved in running buffer and injected into the refractometer with a 1-min injection at 10 μL/min. Regeneration of the system between BlyA injections was achieved by 1-min injections of 0.5% (*w*/*v*) SDS and 40 mM octyl β-D-glucopyranoside, at 10 μL/min. This study was performed at 25 °C, and data were processed using BIAevaluation software (GE Healthcare, Chalfont Saint Giles, UK).

### 5.8. Hemolysis

A total of 100 μL of erythrocyte buffer was pipetted into the wells of the 96-well microtiter plate. BlyA stored in erythrocyte buffer or phosphate buffer (containing 5% glycerol) was assayed. Along the line, we performed 2-fold dilutions of the protein, starting with a concentration of 34 µM down to 0.017 µM. A suspension of bovine erythrocytes in erythrocyte buffer was calibrated to a value of 0.5 at OD630 and 100 μL was added to all wells simultaneously. To follow hemolysis, absorbance was measured at 630 nm. Measurements were performed for 20 min, with one measurement every 20 s.

### 5.9. Sf9 Viability Assay

Sf9 viability assay was performed using CellTiter-Glo reagent (Promega Corp, Madison, WI, USA) according to the manufacturer’s instructions. Briefly, *Spodoptera frugiperda* Sf9 cells were plated in 96-well plates (Costar, Washington, DC, USA) at 3.7 × 10^4^ cells/cm^2^ at 28 °C in protein-free insect cell medium Insect XPRESS with L-glutamine (Lonza, Bend, OR, USA). After 48 h, cells were treated with BlyA (0.156–5 μM) for 30 minutes. Luminescence was measured using a microplate reader (Cytation 3 Imaging Reader; BioTek, Winooski, VT, USA). Data from viability assays are expressed as the percentages of luminescence from treated to untreated cells, as mean ± standard error of two independent experiments, each performed in triplicate.

## Figures and Tables

**Table 1:** Best templates for building the beauveriolysin A protein model.

Short Name	Protein Name Structure ID	Origin	Putative Function	% Identity Residues Coverage Confidence	Partner Protein	Ref.
RahU	RahU protein PDB ID: 6ZC1	*Pseudomonas* *aeruginosa* Proteobacteria	Toxin	32 2–135 97% 100	No data	[37]
PlyA	Pleurotolysin A Membrane embedded pleurotolysin pore with 13-fold symmetry PDB ID: 4V2T	*Pleurotus* *ostreatus* Agaricomycotina	Transport protein	34 2–137 98% 100	Pleurotolysin B; PlyB PDB ID: 4OEJ	[20,23]
IP-1A	Two-component insecticidal protein 16 kDa unit PDB ID: 5V3S	*Alcaligenes* *faecalis* Proteobacteria	Toxin	36 2–137 98% 100	Two-component insecticidal protein 77 kDa unit; IP-1B	[38]
Cry34Ab1	13.6 kDa Insecticidal crystal protein PDB ID: 4JOX	*Bacillus* *thuringiensis* Firmicutes	Toxin	25 4–134 94% 99.1	43.8 kDa insecticidal crystal protein; Cry35Ab1 PDB ID: 4JP0	[39]

The search was performed using a protein sequence of the beauveriolysin A (BlyA) from the fungus *Beauveria bassiana* and the Phyre^2^ web portal for protein modelling, prediction, and analysis [36].

**Table 2:** Best templates for building the beauveriolysin B protein model by a web portal for protein modelling, prediction and analysis.

Short Name	Protein Name Structure ID	Origin	Putative Function	% Identity Residues Coverage Confidence	Partner Protein	Ref.
GNIP1Aa	Gram-negative insecticidal protein PDB ID: 6FBM	*Chromobacterium* *piscinae* Proteobacteria	Insecticidal protein, specifically toxic to *Diabrotica virgifera* *virgifera* larvae upon feeding	13 36–225 38% 95.1	No data	[58]
MPEG-1 (Perforin-2)	Macrophage-expressed gene 1 protein L425K (and w. t.) PDB ID: 6U2W (and PDB ID: 6U23)	*Homo sapiens* Chordata	Immune system	16 11–226 43% 94.6	No	[59]
C9	Complement component C9 PDB ID: 6CXO	*Mus musculus* Chordata	Immune system	14 29–225 39% 93.2	Membrane attack complex	[60]
Plu-MACPF	*Photorhabdus luminescens* MACPF protein PDB ID: 2QP2	*Photorhabdus* *luminescens* Proteobacteria	*Photorhabdus* *luminescens* is a lethal pathogen of insects	11 66–304 47% 93.1	No data	[61]
C8a	Complement component C8 alpha chain PDB ID: 2RD7	*Homo sapiens* Chordata	Immune system	14 36–225 38% 90.7	Membrane attack complex	[62]

The search was performed using a protein sequence of the beauveriolysin B (BlyB) from the fungus *Beauveria bassiana* and the Phyre^2^ web portal for protein modelling, prediction, and analysis [36].

**Table 3:** Organisms with ceramide phosphoethanolamine as the predominant membrane sphingolipid.

Phylum	Organism (CPE + Additional Major Lipid) [25]
Arthropoda, Insecta	*Drosophila melanogaster*, *Musca domestica*, *Aedes aegypti* (+SM), *Culex quinquefasciatus* (+SM), *Manduca sexta* (+SM), *Apis mellifera* (+SM), *Centruroides sculpturatus* (+SM)
Mollusca	*Heterogen longispira* (+CAEP), *Sinotaia histrica* (+CAEP), *Semisulcospira bensoni* (+CAEP), *Batillaria multiformis* (+CAEP)
Ciliophora	*Entodinium caudatum*, *Paramecium tetraurelia*
Apicomplexa	*Toxoplasma gondii*
Euglenozoa	*Trypanosoma brucei*
Metamonada	*Trichomonas vaginalis*
Oomycota	*Pythium ultimum*, *Phytophthora infestans*, *P. capsici*
Bacteroidetes	*Flectobacillus major* (+CPG), *Bacteroides fragilis* (+CPG)

CPE, ceramide phosphoethanolamine; SM, sphingomyelin; CAEP, ceramide aminoethylphosphonate; CPG ceramide phosphoglycerol.

## Data Availability

No new data were created or analyzed in this study. Data sharing is not applicable to this article.

## Supplementary Materials

The following are available online at https://www.mdpi.com/article/10.3390/toxins13110820/s1, Figure S1: Alignments of *Pleurotus ostreatus* aegerolysin protein sequences; Figure S2: Recombinant beauveriolysin A (BlyA) at SDS and blue native PAGE electrophoresis; Figure S3: Circular dichroism spectra of beauveriolysin A (BlyA) at different temperatures; Figure S4: Sedimentation of multilamellar vesicles containing different binary lipid mixtures by beauveriolysin A (BlyA); Figure S5: Hemolysis of bovine erythrocytes by beauveriolysin A (BlyA); Figure S6: Viability assay of insect cells in the presence of beauveriolysin A (BlyA); and Table S1: Description of protein domains position.
