# Supplementary material for: Lipid-Binding Aegerolysin from Biocontrol Fungus Beauveria bassiana
Source: Toxins (Basel). 2021 Nov 20;13(11):820. doi: 10.3390/toxins13110820 (PMC8624791; doi:10.3390/toxins13110820)
Supplement: Supplementary file 1 [file toxins-13-00820-s001.zip › toxins-1442459-supplementary.pdf]

toxins

Supplementary Materials: Lipid-Binding Aegerolysin from Biocontrol Fungus *Beauveria bassiana*

Nada Kraševec, Anastasija Panevska, Špela Lemež, Jaka Razinger, Kristina Sepčić, Gregor Anderluh and Marjetka Podobnik

```
Sequence 1: PleosPC9_1|72745|PlyA(PriA)      Sequences 1:2=99 %     Sequences 2:3=98 %
Sequence 2: sp|P83467|OstreolysinA6          Sequences 1:3=97 %     Sequences 2:4=94 %
Sequence 3: pdb|6MYJ|OstreolysinA6+SM        Sequences 1:4=95 %     Sequences 3:4=93 %
Sequence 4: pdb|4OEB|PleurotolysinA

sp|P83467|OstreolysinA6        -MAYAQWVIIIIHNVGSQDVKIKNLKASWGKLHADGDKDAEVSASNYEGKIVKPDEKLQINACGRSDAAE
pdb|6MYJ|OstreolysinA6+SM      GMAYAQWVIIIIHNVGSQDVKIKNLKASWGKLHADGDKDAEVSASNYEGKIVKPDEKLQINASGRSDAAE
PleosPC9_1|72745|PlyA(PriA)    -MAYAQWVIIIIHNVGSQDVKIKNLKASWGKLHADGDKDAEVSASNYEGKIIKPDEKLQINACGRSDAAE
pdb|4OEB|PleurotolysinA        -MAYAQWVIIIIHNVGSKDVKIKNLKPSWGKLHADGDKDTEVSASKYEGTVIKPDEKLQINACGRSDAAE
                                ****************:********.************:*****:***.::**********.*******

sp|P83467|OstreolysinA6        GTTGTFDLVDPADGDKQVRHFYWDCPWGSKTNTWTVSGSNTKWMIEYSGQNLDSGALGTITVDTLKKGN
pdb|6MYJ|OstreolysinA6+SM      GTTGTFDLVDPADGDKQVRHFYWDSPWGSKTNTWTVSGSNTKWMIEYSGQNLDSGALGTITVDTLKKGN
PleosPC9_1|72745|PlyA(PriA)    GTTGTFDLVDPADGDKQVRHFYWDCPWGSKTNTWTVSGSNTKWMIEYSGQNLDSGALGTITVDTLKKGN
pdb|4OEB|PleurotolysinA        GTTGTFDLVDPADGDKQVRHFYWDCPWGSKTNTWTVSGSNTKWMIEYSGQNLDSGALGTITVDTLKKGN
                               ************************.********************************************
```

Figure S1. Alignment of aegerolysin protein sequences from different *Pleurotus ostreatus* strains. Alignment by ClustalW [92].

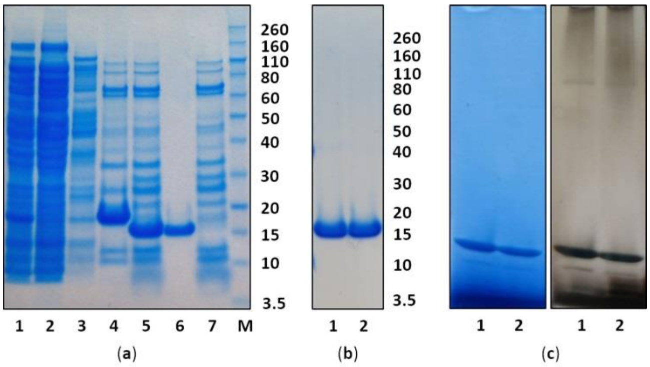

Figure S2. Recombinant beauveriolysin A (BlyA) at SDS and blue native PAGE electrophoresis. (a) Recombinant production of BlyA; different phases of BlyA protein purification followed by Simply blue stained SDS-PAGE gel: 1, bacterial lysate (LB 24 h 20oC); first Ni-NTA chromatography: 2, unbound proteins; 3, non-specifically bound proteins; 4, eluate; 5, eluate after dialysis and TEVcleavage; second Ni-NTA chromatography: 6, purified BlyA protein / unbound proteins after TEV-cleavage; 7, eluate; M, Novex Sharp Unstained Protein Standard. (b) Simply blue stained SDS PAGE gel and (c) Coomassie Brilliant Blue stained and silver stained blue native electrophoresis gel; 1, without the reducent; 2, reduced with DTT.

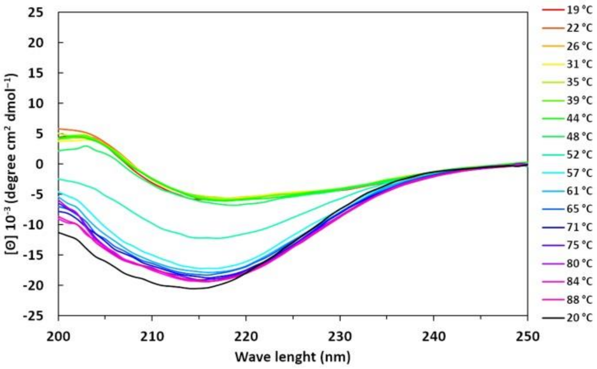

Figure S3. Circular dichroism spectra of beauveriolysin A (BlyA) at different temperatures.

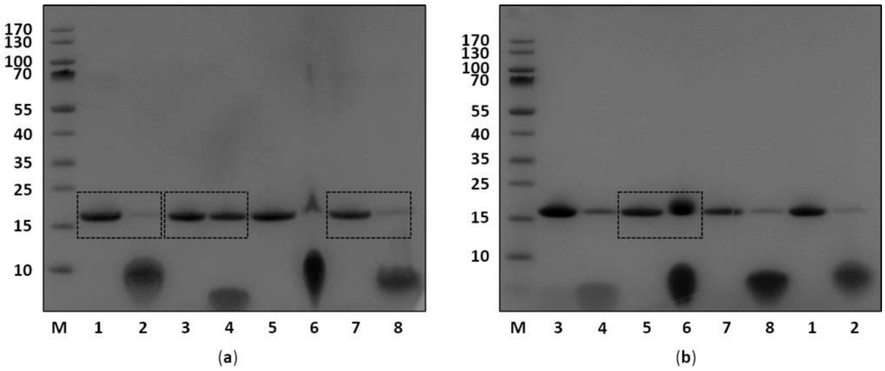

Figure S4. Sedimentation of multilamellar vesicles containing different binary lipid mixtures by beauveriolysin A (BlyA). (a) and (b) Two replicates of the sedimentation assay; M, Thermo Scientific PageRuler prestained protein ladder; 1 and 2, POPC: Chol S and P; 3 and 4, CPE: Chol S and P; 5 and 6, SM: Chol S and P; 7 and 8, CPE: POPC S and P; all lipid mixtures in a 1: 1 molar ratio; MLV, multilamellar vesicles; SM, sphingomyelin; Chol, Cholesterol; CPE, ceramide phosphoethanolamine; POPC, palmitoyl-oleoyl-phosphatidylcholine; S, supernatant; P, pellet.

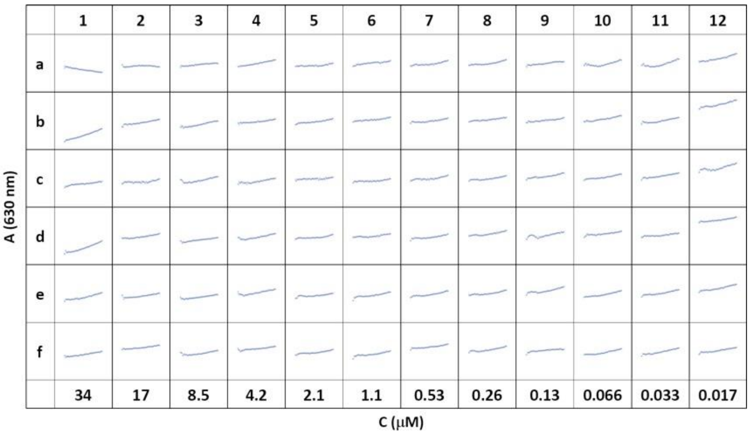

Figure S5. . Hemolysis of bovine erythrocytes by beauveriolysin A (BlyA). Measurements of absorbance at 630 nm were performed in triplicate for 20 min, with reading every 20 s. a-c, BlyA stored in phosphate buffer (50mM NaH2PO4 x 2H2O pH 7,5, 300mM NaCl); and d-f, in erythrocyte buffer (20 mM Trizma base, 140 mM NaCl, pH 7.4).

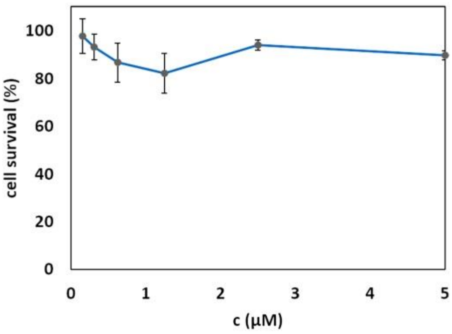

Figure S6. Viability assay of insect cells in the presence of beauveriolysin A (BlyA). Spodoptera frugiperda Sf9 cells were plated in 96-well plates at 3.7 × 104 cells/cm2 at 28 °C in protein-free insect cell medium Insect XPRESS with Lglutamine (Lonza, USA). After 48 hours, cells were treated with BlyA (0.156- 5 µM) for 30 minutes. Data from viability assays are expressed as the percentages of luminescence from treated to untreated cells, as mean ± standard error of two independent experiments, each performed in triplicate.

Table S1. Description of protein domains position.

Protein	Comment	Protein size	Domain	Domain number	Domain position
BlyB	BlyA partner	497			
GNIP1Aa	Model template	536	MACPF	PF01823	62-303
			Vps62	PF06101	326-535
MPEG-1	Model template	642	MACPF	PF01823	108-324
			P2	[58,63]	354-561
C9	Model template	529	TSP_1	PF00090	25-73
			Ldl_recept_a	PF00057	78-113
			MACPF	PF01823	251-485
Plu-MACPF	Model template	511	MACPF	PF01823	130-345
			MABP	IPR023341	359-510
C8a	Model template	367	MACPF	PF01823	142-365
PlyB	Binary partner	523	MACPF	PF01823	91-297
			PlyB_C	PF18684	298-470
IP-1B	Binary partner	703	MACPF	PF01823	78-266
			Insignificant		
Cry35Ab1	Binary partner	383	Ricin_B_lectin	IPR008872	26-147
			Toxin_10	PF05431	174-348

BlyA and B, beauveriolysin A and B; GNIP1Aa, Gram-negative insecticidal protein; MPEG-1, macrophage-expressed gene 1 protein (perforin-2); C9, complement component C9; Plu-MACPF, Photorhabdus luminescens MACPF protein; C8a, complement component C8 alpha chain; PlyB, pleurotolysin B; IP1B, insecticidal two-component protein, 77 kDa unit; Cry35Ab1; 43.8 kDa insecticidal crystal protein. Domains were assigned from EMBL-EBI Pfam [40,64] and some additional domains from InterPro [65] or from reference [63]. MACPF, membrane attack complex/perforin (PF01823); Vps62, vacuolar protein sortingassociated protein 62 (PF06101); β-tripod, β-tripod domain [58]; P2, peripheral domain P2[63]; TSP 1, thrombospondin type 1 domain (PF00090); Ldl recept a, low-density

lipoprotein receptor class A domain (PF00057); MABP, MVB12-associated β-prism domain (IPR023341); PlyB_C, pleurotolysin B C-terminal domain (PF18684); ricin_B_lectin, ricin-type β-trefoil lectin domain (PF00652); toxin 10, insecticidal crystal toxin P42 (PF05431). See Figure 5.
